# Isolation, Characterization and Evaluation of Collagen from Jellyfish *Rhopilema esculentum* Kishinouye for Use in Hemostatic Applications

**DOI:** 10.1371/journal.pone.0169731

**Published:** 2017-01-19

**Authors:** Xiaochen Cheng, Ziyu Shao, Chengbo Li, Lejun Yu, Mazhar Ali Raja, Chenguang Liu

**Affiliations:** College of Marine Life Sciences, Ocean University of China, Qingdao, P. R. China; Massachusetts Institute of Technology, UNITED STATES

## Abstract

Hemostat has been a crucial focus since human body is unable to control massive blood loss, and collagen proves to be an effective hemostat in previous studies. In this study, collagen was isolated from the mesoglea of jellyfish *Rhopilema esculentum* Kishinouye and its hemostatic property was studied. The yields of acid-soluble collagen (ASC) and pepsin-soluble (PSC) were 0.12% and 0.28% respectively. The SDS-PAGE patterns indicated that the collagen extracted from jellyfish mesoglea was type I collagen. The lyophilized jellyfish collagen sponges were cross-linked with EDC and interconnected networks in the sponges were revealed by scanning electron microscope (SEM). Collagen sponges exhibited higher water absorption rates than medical gauze and EDC/NHS cross-linking method could improve the stability of the collagen sponges. Compared with medical gauze groups, the blood clotting indexes (BCIs) of collagen sponges were significantly decreased (*P* < 0.05) and the concentration of collagen also had an influence on the hemostatic property (*P* < 0.05). Collagen sponges had an improved hemostatic ability compared to the gauze control in tail amputation rat models. Hemostatic mechanism studies showed that hemocytes and platelets could adhere and aggregate on the surface of collagen sponge. All properties make jellyfish collagen sponge to be a suitable candidate used as hemostatic material and for wound healing applications.

## 1. Introduction

Uncontrolled hemorrhage after trauma and in surgical procedures has associated with the increased mortality rate, and emergency hemostatic management has been a crucial focus [1–3]. The body’s natural responses to an injury are comprised of hemostatic process and healing of the wound site [4]. However, the body's natural mechanism is unable to control massive hemorrhaging caused by major trauma or surgery. There is a medical need to develop an effective hemostat for emergency circumstances [5].

Traditional hemostasis techniques (cautery and suture ligation) used in the operating room can always cause problems such as oozing bleeding [3] and damaging of capillaries resulting in tissue necrosis [6]. For these reasons, a number of hemostatic agents that can arrest bleeding and promote hemostasis have been developed. Either natural or synthetic polymers have been employed for the construction of hemostatic agents. The configurations of these agents are mostly sheet [7], sponge [8–9] and glue [10]. Natural polymers have been widely used as topical hemostatic agents for their excellent properties such as biodegradability and biocompatibility.

Natural hemostatic agents such as oxidized cellulose [11–12], chitosan [9,13–14], gelatin [15], thrombin [16], collagen [17–18] and fibrin [19] can be divided in active and passive agents [20]. For example, fibrin glue has been widely used as active hemostatic agents which can effectively prevent the postoperative complications such as bleeding, hematoma formation, seroma, edema, and prolonged drainage [21–23]. However, plasma derived fibrin sealant/hemostatic products can cause viral contamination and anaphylaxis [24–25]. Horowitz and Busch have reported that the fibrin sealants have some risks of transmission of HIV, hepatitis virus and other parvovirus [26]. Passive hemostatics, including collagen, gelatin and oxidized cellulose, are not biologically active; their mechanism of action is to provide platelet activation and aggregation [3]. Among them, collagen has been reported as useful hemostatic agent [27–30]. Collagen is the most abundant protein (approximately 30% by weight of total protein) in human’s body which consists in extracellular matrix [31]. The triple helical structures exist in all collagen molecules and form three parallel, left-handed helical polypeptide α-chains [32]. Each α-chain has a characteristic [Gly-X-Y] domain repeat, in which X and Y mostly represent the proline (Pro) and hydroxyproline (HyP) [33]. Moreover, it has been proved that collagen has better biocompatibility, higher biodegradability, lower antigenicity and cell-binding properties as a natural protein, which can be degraded into physiologically tolerable compounds in vivo. [34–36]. The role of collagen in regulation of hemostasis is that collagen can combine with thrombin to stimulate the platelets and promote the release of clotting factors [37], and can also induce platelets aggregate formation [38].

Commercially available collagen-based agents are usually derived from bovine and swine. Collagen of bovine origin is associated with transmission of bovine spongiform encephalopathy (BSE) and transmissible spongiform encephalopathy (TSE) [39–40]. Porcine collagen can also cause religious problems in some regions. So, it is necessary to obtain a much safer collagen from ocean environment in order to find an alternative resource. Jellyfish has been shown to be rich in minerals and proteins [41], and collagen is a major protein in jellyfish [42]. China is the first country to process the jellyfish as food and medicine and Chinese have been eating jellyfish for more than a thousand years [43–44]. One of the most abundant species of jellyfish in China is *Rhopilema esculentum* Kishinouye (*R*. *esculentum*), and it is widely distributed over the South China Sea, the Yellow Sea and Bohai Sea [45]. Liu et al. [46] reported that the peptides derived from *R*. *esculentum* could reduce the blood pressure in spontaneously hypertensive rats and be used as antihypertensive compounds in functional foods. Yu et al. [47] reported that proteins isolated from jellyfish *R*. *esculentum* showed strong antioxidant activity and might be applied in the food and pharmaceutical industries.

To the best of our knowledge, so far, no report has been published on the hemostatic properties of collagen from the jellyfish *R*. *esculentum*. In the present paper, we extracted collagen form jellyfish mesoglea, and the characters of collagen were studied. Furthermore, we prepared collagen sponge and the structure of the sponge was analyzed by Fourier Transform Infrared Spectrum (FITR) and Scanning Electron Microscope (SEM). Finally, the hemostatic ability of jellyfish collagen sponge was assessed by whole blood clotting and rat tail amputation experiments.

## 2. Materials and Methods

### 2.1 Ethics statement

As a normal kind of jellyfish, no specific permits are required for the studies on *R*. *esculentum* so far (There was no specific permissions were required for catching jellyfish). This study was carried out in strict accordance with the recommendations in the Guide for the Care and Use of Laboratory Animals (2011) and the Guide for the Use of Experimental Animals of Ocean University of China. The protocols for animal care and handling were approved by the Institutional Animal Care and Use Committee of Ocean University of China (Permit Number: 20130601).

### 2.2 Materials

The jellyfish *R*. *esculentum*, caught in the Yellow Sea of Qingdao, China, was kept in cold water (There was no specific permissions were required for these activities). After it transported to the laboratory, mesoglea, the major part of jellyfish umbrella was manually excised *in vivo* and cut into pieces. Then the samples were immersed in cold distilled water at 4°C for 3 days, and the water was changed two times every day to desalting. After that, the washed samples were stored in polyethylene bags and frozen in −80°C until further used. Sprague-Dawley (SD) rats used in this study (2 to 3 months old, weighting 250 ± 50 g) were purchased from Qingdao Experimental Animal Center (China).

1-ethyl-3-(3-dimethylaminopropyl) carbodiimide (EDC), and N-hydroxysuccinimide (NHS) were purchased from Sigma Chemical Co. (MO, USA). All other reagents used in this paper were of analytical grade and obtained from Sinopharm Chemical Reagent Co., Ltd (Shanghai, China).

### 2.3 Extraction of collagen from jellyfish

The collagen from jellyfish mesoglea was extracted according to previously published method with a little modification [42]. Briefly, mesoglea pieces were smashed by tissue homogenate machine (IKA T10 Basic ULTRA-TURRAX, Staufen, Germany). The smashed mesoglea was added into 0.6 M acetic acid solution with continuous stirring at 4°C for 72 h. The mixture was filtered through cheesecloth to remove water-insoluble components. Then solid NaCl was added into the filtrate to a final concentration of 0.9 M and the precipitate was harvested by centrifugation at 4,000 *g* for 15 min. After centrifugation, the precipitate was named as acid-soluble collagen (ASC). The pepsin-soluble collagen (PSC) was prepared by dispensing ASC into 20 volumes of 0.5 M acetic containing 1% pepsin (w/w, EC 3.4.23.1, Sigma, USA). After incubation at 4°C for 24 h, the mixture was centrifuged at 10,000 *g* for 30 min and supernatant was dialyzed against 0.02M Na_2_HCO_3_ to inactive the pepsin, and PSC was salted out by 0.9 M NaCl solution. The ASC and PSC were dissolved in 0.5 M acetic acid solution and dialyzed against deionized water at 4°C for 72 h to get the collagen solution. Finally, the collagen fibers were obtained by lyophilization.

### 2.4 SDS-polyacrylamide gel electrophoresis (SDS-PAGE)

SDS-PAGE was performed as previously described [48]. The extracted jellyfish collagen was dissolved in the 0.02 M sodium phosphate (pH 7.2) containing 10 g/L SDS and 3.5 M urea. The stacking and separation gels concentrations were 5% and 8% respectively. After stained by Coomassie Brilliant Blue R250, the bands of ASC and PSC were contrasted with type I collagen from murine tail tendon.

### 2.5 Amino acid analysis

After protecting the cysteine, methionine and tyrosine, acid-soluble collagen was hydrolyzed by acid such as 6 N hydrochloric acid in vacuum or N_2_ at 110°C for 10-24h. Hydrolysates of PSC were characterized by amino acid analyzer (HITACHI L-8900 Amino Acid Analyzer, Tokyo, Japan).

### 2.6 Preparation of the hemostatic sponges

Jellyfish collagen was immersed in acetic acid solution prepared in different ratios (2.5, 3.3 and 5 mg/ml) and stirred constantly for about 20 h after the collagen was totally dissolved. The collagen solutions were dialyzed against deionized water at 4°C for 72 h. The resulted solutions were added into 12-well plates (3 ml per well) and lyophilized at -50°C to fabricate the collagen sponges. The collagen sponges were cross-linked by different concentrations of EDC/NHS (EDC: 25 mM, 50 mM, and 100 mM; NHS: 6 mM) dissolved in 95% ethanol for different time periods (12 h and 24 h) at 4°C. After cross-linking, the sponges were washed five times by deionized water and lyophilized.

### 2.7 Determination of the degree of cross-linking

The degree of cross-linking was determined by ninhydrin assay which could measure the percentage of free amino groups remaining in the collagen sponges before and after cross-linking. In the ninhydrin assay, lyophilized jellyfish collagen sponges were weighted and 5 mg samples were mixed with 2 ml ninhydrin solution and heated to 100°C in water bath for 20 min and then cooled down to room temperature. The solution was added into 10ml 50% isopropanol and the optical absorbance at 570nm (Abs_570_) was measured by spectrophotometer (Shimadzu UV-3600Plus, Kyoto, Japan). The amount of free amino groups is proportional to the value of Abs_570_, and glycine at various known concentrations was used to create standard curve of glycine concentration vs absorbance. The degree of cross-linking of sample is then calculated following the equation:

$$Degree\;of\;cross-linking\;=\;\frac{{Amino}_{0}-{Amino}_{c}\;}{{Amino}_{0}}\times 100$$

Where *Amino*_*0*_ is the free NH_2_ concentration in non-cross-linked samples, and *Amino*_*c*_ is the free NH2 concentration in cross-linked samples.

### 2.8 Scanning electron microscope (SEM) analysis

In order to study the morphology and internal structure of the sponges, the JSM-840 scanning electron microscope (JEOL JSM-840 Scanning Microscope, Tokyo, Japan) was used. After the sponges were coated with platinum, the surface and cross-section of the sponges were examined by SEM.

### 2.9 Infrared (IR) spectrum

Collagen IR spectrum measurements were carried by infrared spectrometer (Thermo Scientific Nicolet iS5 Infrared Spectrometer, MA, USA). The collagen sponges were grounded to powder with KBr (Spectral pure, Sinopharm Chemical Reagent Co., Ltd, Shanghai, China), and the mixture was pressed to films. The main parameters of IR setting were the resolution for 4 cm^-1^ and the scan number for 64 times and the SNR for greater than 25,000. The spectrum between 4000–400 cm^-1^ was recorded.

### 2.10 Water absorption capacity (WAC) measurement

A gravimetric method was carried out to determine the water absorption capacity of collagen sponges [49]. Different sponges were cut into 10×10 mm^2^ pieces and were weighed at dry status (*W*_*d*_); these pieces were then immersed in the distilled water at room temperature and water on the surface was removed with the help of filter paper. The swollen membranes weights were measured (*W*_*s*_). Water absorption capacity (WAC) of sponges was calculated by the following equation:

$$WAC\;=\;\frac{W_{s}-W_{d}}{W_{d}}$$

### 2.11 Degradation rate

Collagen sponges divided into different groups were weighed in the dry state as the initial weight (*W*_*i*_). And these sponges were immersed in the distilled-water and incubated at 37°C for 3 days. The final dry weights of the membranes (*W*_*f*_) were measured to calculate the membrane degradation using the following equation:

$$Weight\;loss\;=\;\frac{W_{i}-W_{f}}{W_{i}}$$

### 2.12 Cell culture and viability

The mouse fibroblasts (L-929) cells were obtained from American Type Culture Collection (ATCC Number: CCL-1), and the cells were maintained in Dulbecco's Modified Eagles Medium (DMEM) with 10% fetal bovine serum (FBS) supplemented with penicillin (120 U/ml) and streptomycin (75 mg/ml) at 37°C with 5% CO_2_. When the cells reached 80%-90% confluency the cells were treated with trypsin for passing. The sterilized sponges were put into 24-well plates, and the cells were seeded on the microparticles with the seeding density of 3×10^4^ cells/well. The polystyrene was used as control. Cell viability determined by evaluating the uptake of 3-(4,5-dimethylthiazol-2-yl)-2, 5-diphenyl-tetrazolium bromide (MTT) by the cells. Briefly, after incubation for 7 days, the medium was removed and 500 μL MMT (5 mg/ml, soluted in DEME) was added into the wells. The sponges were washed with PBS after incubated for 3 h in 37°C and were transmitted into other plates containing DMSO. And the Abs_550_ of the solutions were measured.

$$\%\;of\;control\;=\;\frac{{Abs}_{550}\;of\;sample}{{Abs}_{550\;}of\;control}\times 100$$

### 2.13 Whole blood clotting

Collagen sponges were placed into polypropylene tubes, and pre-warmed to 37°C. Citrated whole blood (0.2 ml) was then dispensed onto the sponges, and 20 μl of 0.2 M CaCl_2_ solution was added to start coagulation. The tubes were incubated at 37°C and shaken at 30 rpm. After 5 min, red blood cells (RBCs) that were not trapped in the clot were hemolyzed with 25 ml of deionized water. The absorbance of the resulting hemoglobin solution was measured at 540 nm (*D*_*s*_), and the absorbance of 0.2 ml whole blood hemolyzed with 25 ml deionized water at 540 nm was denoted as *D*_*0*_. The blood clotting index (BCI) was calculated with the formula: BCI = *D*_*s*_/*D*_*0*_.

### 2.14 Interaction between the collagen sponges and blood cells

In order to explore the hemostatic mechanism of jellyfish collagen and evaluate the hemostatic effect of collagen sponges, the morphology study and adhesion of RBCs and platelets on the collagen sponges were carried by SEM. The collagen sponge (1 × 1 cm^2^) was immersed in PBS (pH 7.4) at 37°C for 2 h, and 0.1 ml citrated whole blood was added onto the sponges (n = 5). The sponge containing whole blood was incubated at 37°C for 1 h. The sample was then gently washed three times with PBS (pH 7.4) and fixed with 2.5% glutaraldehyde at 4°C for 2 h. After dehydrated with 50, 60, 70, 80, 90 and 100% ethanol for 10 min orderly, the sample was lyophilized for SEM observation.

### 2.15 Platelet adhesion

Platelet adhesion on collagen sponges was observed by fluorescence staining with calcein-AM. The whole blood was centrifuged at 300 *g* for 20 min at 4°C to obtain platelet-rich plasma (PRP), and PRP was added to 24-well plate (1 ml/well) and incubated with collagen sponges at 37°C for 1 h with gentle shaking. Then the sponges were rinsed three times with PBS to remove non-adhered platelets. The adhered platelets were stained with 2 μM calcein-AM PBS solution. The stained platelets were observed under fluorescence microscope (Olympus CX23, Japan), and the numbers of adhered platelet were counted.

### 2.16 Murine tail amputation

Surgeries were completed on 2 to 4-month-old Sprague-Dawley rats (200–300 g). Anesthesia was induced with 4% isoflurane, and maintained with 2.5% isoflurane by anesthetic ventilator. In addition, all efforts were made to minimize suffering. Tail amputation at 50% tail length was completed using surgical scissors. After the amputation, the pre-weighted materials (n = 7 for each group) were immediately put on the wound with minimal pressure and after 5 min the mass of blood loss was recorded. In an analogous experiment, time to hemostasis was recorded after checking for bleeding in 1 min intervals. After the tail amputation, the condition of rats used in this study were monitored in every 12 h, and pentobarbital sodium was used to minimize suffering of the rats. When the experiment was finished, none of the experimental rats was died during the study, and finally the euthanasia was induced with 70% carbon dioxide by anesthetic ventilator.

### 2.17 Statistical analysis

Statistical analysis was performed by analysis of variance (ANOVA) and Kruskal-Wallis test using SPSS 17.0 package (SPSS Inc., IL, USA). Statistical significance was determined at a value of *P* < 0.05.

## 3. Results and Discussion

### 3.1 Characterization of jellyfish collagen

#### 3.1.1 Yield of ASC and PSC from jellyfish mesoglea

The yields of collagen extracted with acid and pepsin were 0.12% and 0.28% respectively (on a wet weight basis). The higher yield was obtained with the pepsinized extraction method. This may be attributed to pepsin cleaving the peptides localized at the telopeptide region, resulting in the increased collagen extraction efficacy [50–51]. This result was in agreement with other reports which reported that higher yields of PSC were also found in four jellyfish species (*Aurelia aurita*, *Cotylorhiza tuberculata*, *Pelagia noctiluca*, and *Rhizostoma pulmo*) [52], scale [53] and skin [54] of fish.

#### 3.1.2 SDS-PAGE patterns

**Fig 1:**
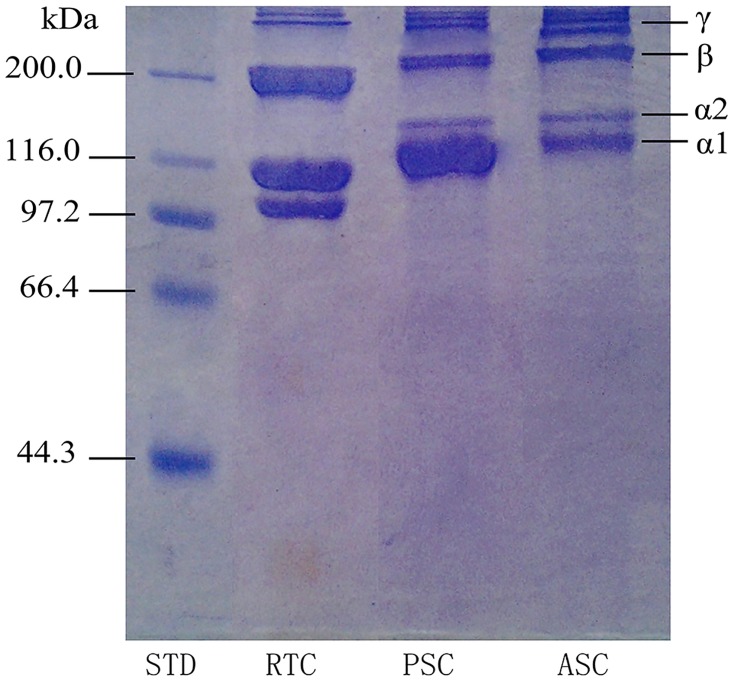
SDS polyacrylamide gel electrophoresis pattern of collagens from jellyfish mesoglea. STD: molecular weight marker; RTC: rat tail type I collagen; PSC: pepsin soluble collagen; ASC: acid soluble collagen.

Electrophoresis analysis of ASC and PSC from jellyfish mesoglea was shown in Fig 1. The patterns revealed that both ASC and PSC consisted of α1 and α2 chains around 116 KD with a ratio of 2:1, and the β chains (dimers), γ chains (tripolymers) and cross-linked α chains were located on high molecular mass region (above 200 KD). It had been reported that the collagen isolated from marine invertebrate animals, such as Red Sea cucumber (*Stichopus japonicus*) [55], sea urchin (*Asthenosoma ijima*) [56], and starfish (*Asterias amurensis*) [57], was classified into type I collagen. Moreover, the marine vertebrate collagen, such as scales and skin of fish also belongs to type I collagen [58–60]. SDS-PAGE profiles of ASC and PSC from jellyfish showed a similar pattern, demonstrated that the ASC and PSC extracted from jellyfish mesoglea were similar to type I collagen. Furthermore, β chains (dimers) and γ chains (tripolymers) were observed in both ASC and PSC. Compared with ASC, the band intensities of β chains and γ chains of PSC were weaker and the band intensity of α chain was obviously stronger. This result indicated that the inter- and intra-crosslinking of collagen molecular was destroyed by pepsin to some extent [51], and β chains and γ chains degraded to α chains, which suggested that α chain was the basic building block in collagen. As exhibited in the SDS-PAGE pattern, we also found that the molecular weight of α and β chains from jellyfish collagen were slightly higher, which revealed the structural differences between jellyfish collagen and collagen from terrestrial vertebrates. According to the modern point of view, the evolution of fibrillar collagen genes is the major reason which causes these structural differences [61].

#### 3.1.3 Amino acid composition

Amino acid composition of PSC from jellyfish, expressed as residues per 1000 total residues, is shown in Table 1. The most abundant amino acid was glycine (Gly) in jellyfish collagen (277.8 residues/1000 residues), similar to the calf-skin collagen (325.6 residues/1000 residues). In general, glycine spaced in every third residue in collagen except the first 10 amino at C-terminus region and the last 14 amino at N-terminus region [62], so glycine accounts for about one-third of total residues. The alanine (Ala) content in PSC from collagen was high (108.6 residues/1000 residues). Besides, asparagine/aspartic acid (Asp), glutamine/glutamic acid (Glu) and arginine (Arg) were also found at high content in PSC. The proline (Pro) in PSC from jellyfish was 72.3 residues/1000 residues, which was lower than that from fish scale (108 residues/1000 residues) [63], fish skin (112 residues/1000 residues) [64], and body wall of sea cucumber (95 residues/1000 residues) [65]. The imino acids, which include proline and hydroxyproline, can contribute the stability of collagen because the triple helix of collagen is held by the hydrogen bonds between hydrogen atoms and pyrrolidine rings of these imino acids [66]. Moreover, the content of cysteine (Cys) was negligible in PSC (2.8 residues/1000 residues). Lysine (Lys) content in PSC was 51 residues/1000 residues, higher than the collagen extracted from scales of spotted golden goatfish [19] and calf skin type I collagen [67]. It is reported that lysine and hydroxylysine exist in cross-linking of telopeptide of collagen molecules [50]. This result revealed that the β chains and γ chains in PSC from jellyfish were more abundant. PSC from jellyfish can be classified as type I collagen which is similar to calf skin collagen. However, there were some slight differences between the amino acid composition of typical type I collagen and jellyfish collagen, which led to the structural changes of jellyfish collagen.

**Table 1:** Amino acid composition of the collagen from jellyfish (PSC) and calf-skin collagen (results are expressed as residues/1000 residues).

Amino Acid	PSC	Calf-skin collagen
Asp	68.3	49.3
Thr	36.5	23.5
Ser	44.4	40.9
Glu	85.8	78.4
Gly	267.9	325.6
Ala	108.6	129.1
Cys	2.8	0.9
Val	38.0	39.7
Met	11.6	8.6
Ile	30.5	23.5
Leu	41.9	38.2
Tyr	18.3	9.1
Phe	29.6	19.4
His	5.7	0.6
Lys	51.0	42.7
Arg	76.9	53.6
Pro	72.3	116.9
Trp	0.0	0.0
TOTAL	1000	1000

### 3.2 Characterization of jellyfish collagen sponges

#### 3.2.1 Degree of cross-linking

In this study, 1-ethyl-3-(3-dimethylaminopropyl) carbodiimide (EDC), a non-toxic cross-linking reagent, was used to cross-link jellyfish collagen to improve the mechanical property of collagen sponges. As shown in Table 2, under the same EDC concentration (50 or 100mM), the cross-linking degree increased with increasing cross-linking time. Similarly, the cross-linking degree increased with increasing EDC concentration with the same time (12 or 24h). However, jellyfish collagen concentration had no significant impact on the cross-linking degree (*P* > 0.05). When the sponges were cross-linked by 100mM EDC for 24h, 90% of the amino groups in both test membranes were cross-linked, which indicated that EDC is an effective cross-linking reagent.

**Table 2:** Cross-linking degree of jellyfish collagen sponges.

EDC concentration (mM)/ Cross-linking time (h)	Collagen concentration (mg/ml)	
	2.5	3.3
	Degree of Cross-linking	
50/12	35.17±4.35	36.61±8.65
100/12	48.76±2.89	46.73±7.41
50/24	64.78±2.13	65.14±2.97
100/24	86.92±4.87	88.17±5.67

#### 3.2.2 Fourier transform infrared spectroscopy

**Fig 2:**
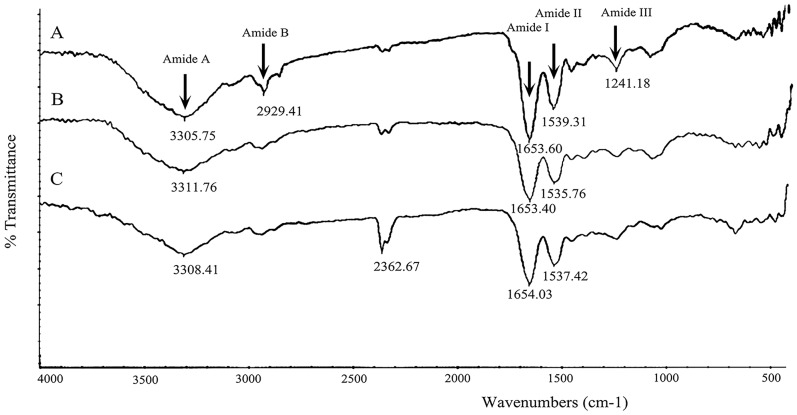
Fourier transform infrared spectrum of different collagen sponges. A, uncross-linked collagen sponge; B, collagen sponge cross-linked with 50mM EDC for 12h; C, collagen sponge cross-linked with 100mM EDC for 24h (The unit of the numbers in figure is cm^-1^).

The FITR spectrum of collagen sponges with different cross-linking degree is shown in Fig 2. The main bands of collagen arisen from peptide bond vibrations are amide A, I, II and III. It is reported that N-H stretching vibrations, which represent amide A band, usually occurred at 3400 cm^-1^ to 3340 cm^-1^ [68]. In this study, the amide A band position of different collagen sponges was found at ~3300 cm^-1^, which shifted to a lower frequencies. This may be because the N-H groups of collagen are involved in a hydrogen bond [69]. The amide I band of collagen sponges was found at ~1650 cm^-1^, and this was the carbonyl group (C = O) stretching vibration coupled with COO^-^ [70]. The difference between uncross-linked and cross-linked collagen sponges in the amide I band indicated that the C = O bond in collagen was slightly weakened because of the formation of new bonds between carboxyl groups and amine groups [71]. Peaks at ~1530 cm^-1^ are the amide II bands in all groups of collagen sponges, which is N-H bend coupled with C-N stretching vibration [72]. The position of amide III band was found at ~1240 cm^-1^ [70], which is the evidence of the existence of helical structure [73]. However, the amide III bands of cross-linked collagen sponges (Fig 2B and 2C) were much weaker than that of untreated collagen sponge. This demonstrated that the cross-linking process by EDC influenced the structure of collagen fibres.

From Fig 2, we could also observe the band at ~2930 cm^-1^ arising from C-H stretching of amide B [74] was very weak in cross-linked collagen sponges (Fig 2B and 2C). The modification can be explained by the conformational changes of the secondary structure of collagen after cross-linking [75]. It was noticed that the intensity of band at 2362.67 cm^-1^ increased with the enhancement of degree of cross-linking. This may be caused by the tertiary amine (N-H^+^ groups) in *o*-acylisourea as an intermediate [76].

#### 3.2.3 Morphology of the collagen sponges

**Fig 3:**
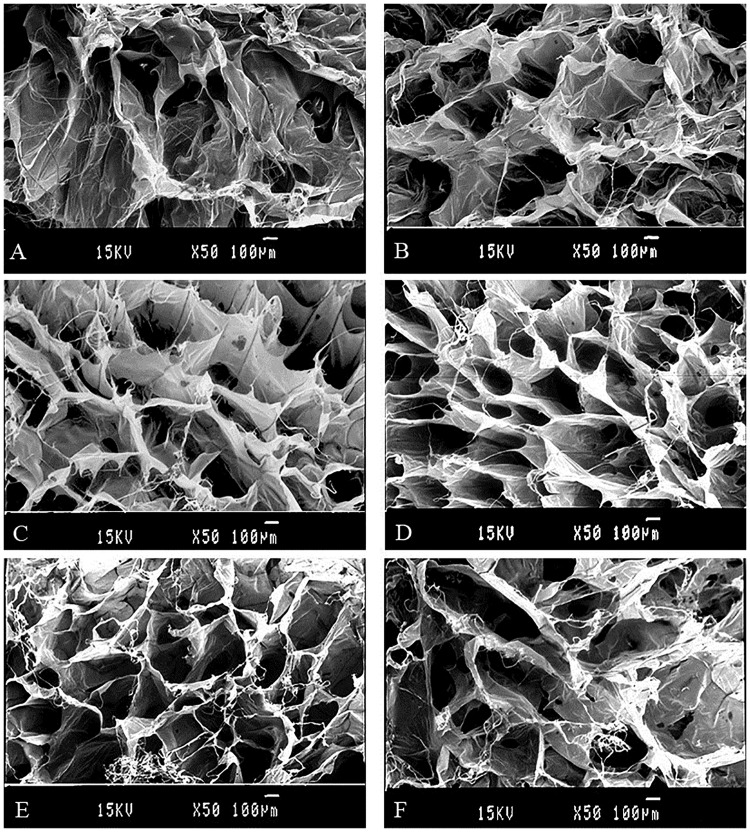
SEM images of cross-linked and uncross-linked lyophilized collagen sponges of different concentration. A, B: cross-section and surface of 3.3 mg/ml collagen sponges cross-linked by 100mM EDC for 24 h, respectively. C, D: cross-section and surface of 10.0 mg/ml collagen sponges cross-linked by 100mM EDC for 24 h, respectively. E, F: cross-section and surface of 10.0 mg/ml collagen sponges uncross-linked, respectively.

Porosity and pore size are considered as important factors in sponges when used as hemostat. The collagen sponges were prepared by lyophilization of jellyfish collagen solution. The microstructure of sponges was observed by scanning electron microscope (SEM). From Fig 3, we found interconnected network of pores in the sponges, which insure that sponges have excellent water-absorbing properties. With the increasing concentration of the collagen solution the porosity and pore size showed a tendency of decline (Fig 3A, 3B, 3C and 3D). Compared with uncross-linked ones, the cross-linked sponges did not show significant differences (Fig 3C, 3D, 3E and 3F). We could draw the conclusion that cross-linking process does not influence the microstructure apparently, and the result was in agreement with Song and his coworkers who reported that EDC/NHS cross-linking density did not significantly influence the microstructure of collagen scaffolds [67].

#### 3.2.4 Water absorption capacity of collagen sponges

**Fig 4:**
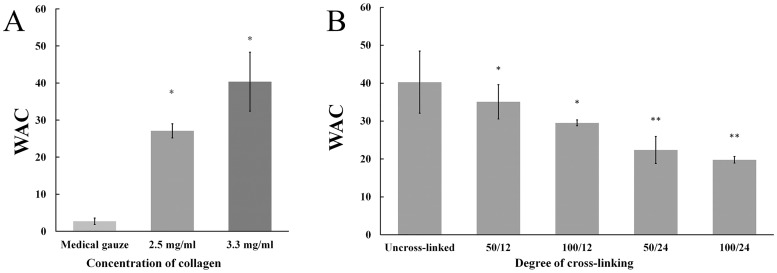
Water absorption capacity (WAC) of different sponges. (A) shows the WAC of different collagen sponges (3.3 mg/ml and 2.5 mg/ml), and medical gauze group was set as control. The symbol (*) denotes statistical significance from control group (*P* < 0.05). (B) shows the sponges with different cross-linking degree fabricated by 3.3 mg/ml collagen have different WAC. Degree of cross-linking is expressed as EDC concentration (mM)/cross-linking time (h) (50/12, 50/24, 100/12 and 100/24 in this study). The symbol (*) and (**) denotes statistical significance from uncross-linked groups and 100/12 groups, respectively (*P* < 0.05).

Water absorption capacity reflects the blood-absorbing property of hemostat, and it is an important parameter in evaluation of hemostat and wound dressing materials [77]. Fig 4A revealed that collagen sponges exhibited higher water absorption rates than medical gauze (*P* < 0.05) and collagen sponges fabricated with high level concentration of collagen solution possess higher WAC (More information see in S1 and S2 Tables). This may be due to porous structure of collagen sponge and the water retention of collagen fibers. Fig 4B showed the effect of cross-linking degree on the WAC of collagen sponge. The concentration of EDC and cross-linking time represent the degree of cross-linking. The water absorption rate decreased with increasing concentration of EDC and prolonging cross-linking time. From the results, collagen sponges with a higher degree of cross-linking revealed a lower WAC value. These results suggested that with increasing the cross-linking degree and time the collagen sponges became more rigid and thus reduced the swelling of the sponges.

#### 3.2.5 Degradation rate in vitro

**Fig 5:**
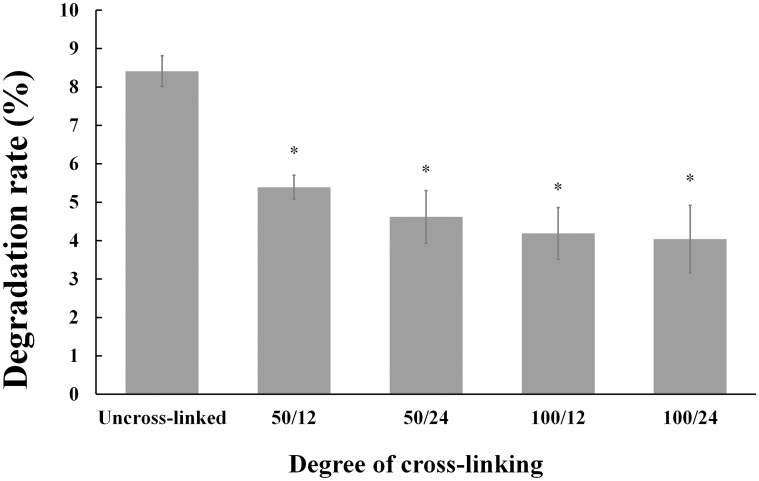
Comparison of weight loss of uncross-linked collagen (Control group) and cross-linked collagen groups. Degree of cross-linking are expressed as EDC concentration (mM)/cross-linking time (h) (50/12, 50/24, 100/12 and 100/24 in this study). The symbol (*) denotes statistical significance from control group (*P* < 0.05).

Fig 5 showed the weight loss of collagen sponges after putting in distilled water for 3 days. From the results, it was observed that after 3 days the collagen sponges lost about 8% weight, while the cross-linked collagen sponges lost up to 4% ~ 5% weight. Moreover, the untreated collagen sponges became fragmented and the shape of cross-linked collagen sponges maintained very well through the whole experiment. The slow degradation and improved mechanical property of cross-linked collagen sponges might be attributed to the EDC/NHS cross-linking between carboxyl groups and amine groups of collagen sponge. Collagen sponges serve as a matrix for clot formation and stable collagen sponge can improve platelet aggregation, degranulation, and release of clotting factors. The weight loss results suggested that EDC/NHS crosslinking was a useful way to improve the stability of the collagen sponge. From these results, including water absorption and degradation rate, we found that the cross-linking process had a similar effect on the jellyfish collagen compared to the collagen from other sources [78]. This maybe because the jellyfish collagen has the typical structure in which there were water fixation sites and the bonds produced by cross-linking plays an important part in the stabilization of collagen [79].

### 3.3 Cytotoxicity study

**Fig 6:**
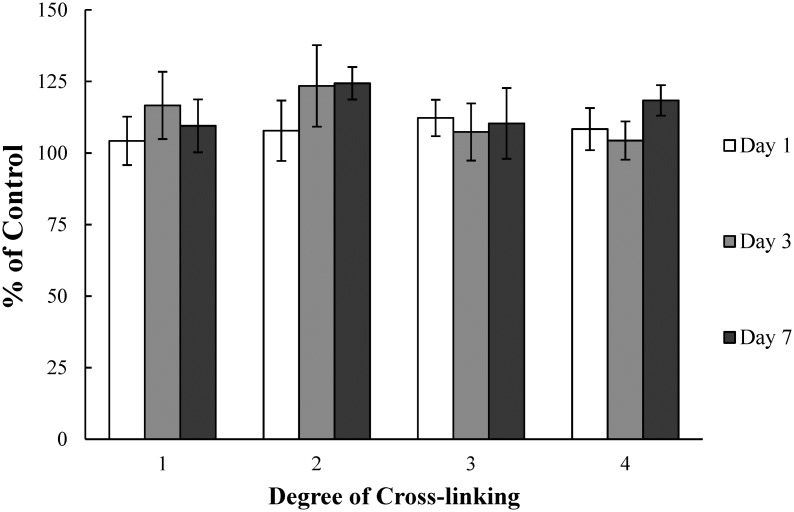
Viability of cells cultured in direct contact with various biomaterials at 1, 3 and 7 days, as determined by MTT assay. Viability is expressed as a percentage of live cells compared to positive control cells: (1) uncross-linked 2.5 mg/ml collagen sponges; (2) 2.5 mg/ml collagen sponges cross-linked with 100 mM EDC for 24h; (3) uncross-linked 3.3 mg/ml collagen sponges; (4) 3.3 mg/ml collagen sponges cross-linked with 100 mM EDC for 24h.

The in vitro cytotoxicity of collagen sponges was evaluated using fibroblast by MTT assay, which can present the parameter of metabolic activity [80, 81]. Viabilities of the cells cultured on the collagen sponges are showed in Fig 6. In this study, relative cell viability in this study is expressed as the Abs_550_ value of cells on the sample versus that of cells on tissue culture plates. From the result, we could found that jellyfish collagen did not induce a significant cytotoxic effect (*P*>0.05) and the cells cultured on sponges showed much higher viability than tissue culture plates. The values of ‘% of control’ were all above 100 which indicated that the jellyfish collagen could promote growth and viability of fibroblast. Moreover, the cell viabilities exhibited no significant difference between day 1, 3 and 7, and the viability of fibroblasts did not decrease within the 7-day observation period. In comparison, the cells cultured on the collagen sponges with different degree of cross-linking showed no significant differences (*P*>0.05). This revealed that the method of cross-linking with EDC was less likely to induce any significant adverse effect on the viability of cells.

### 3.4 Interaction between the collagen sponges and blood cells

**Fig 7:**
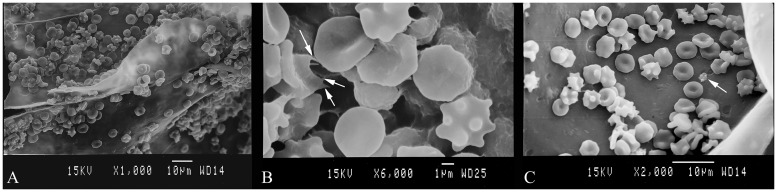
SEM images of blood cells and platelets adhered on the surface of collagen sponges. (A) Aggregation and morphology of RBCs on the collagen sponge. (B) RBCs generated spiny pseudopodia (arrow). (C) Platelet adhered on the collagen sponge exhibited regular resting shape (arrow).

The morphology of RBCs and platelets, and their adhesion on the collagen sponges were observed by SEM. To reveal whether the collagen sponges could impact the physiological action during the coagulation process, the whole blood was added onto the sponges with RBCs and platelets to have an immediate contact with the surface of collagen sponge. Fig 7A showed the SEM image of blood cells adsorbed on the surface of collagen sponges. A mass of RBSs adhered to the surface of sponge and the aggregation of RBCs was obviously on collagen. Under high magnification, as shown in Fig 7B, the morphology of aggregated RBCs changed to irregular shape. Furthermore, it was found that some RBCs stretched out spiny pseudopodia (arrows in Fig 7B). The above mentioned phenomena revealed that the blood clot was formed on the surface of collagen sponge and RBCs went through physiological changes caused by collagen sponge. Fig 7C showed the platelets adhered to the surface of sponge and only a few of platelets with regular resting shape were found adhering on the surface of sponges.

**Fig 8:**
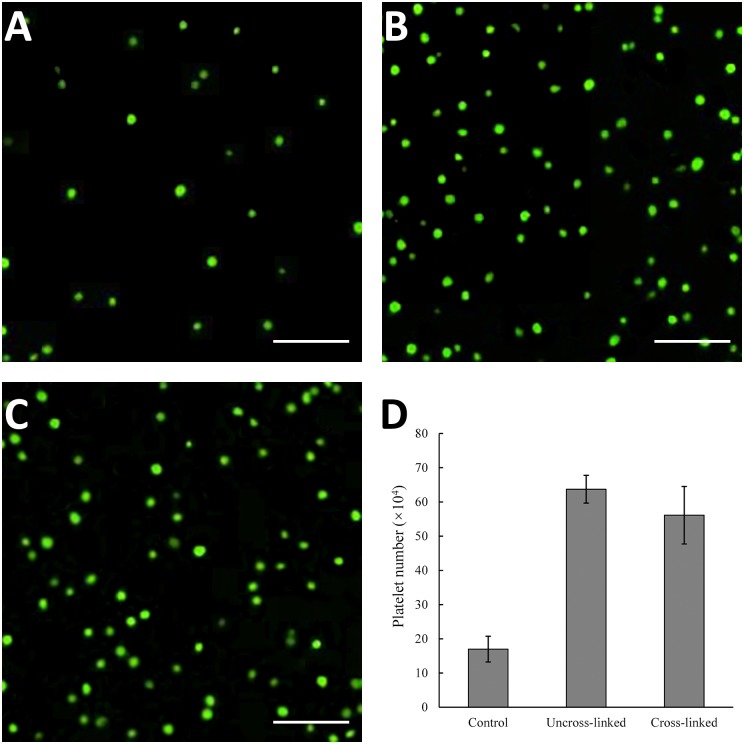
Collagen sponges effect on platelets adhesion. The adherent platelets on different sponges were observed by calcein-AM staining, and images were showed in (A) control group, (B) uncross-linked collagen sponges and (C) cross-linked collagen sponges. The numbers of platelets were counted (D). Scale bar, 30 μm.

The platelets adhered on collagen sponges were stained with calcein-AM, and observed under fluorescence microscope. The results were showed in Fig 8. It was observed that platelets adhered on both uncross-linked and cross-linked collagen sponges were more when compared with control group. Moreover, the numbers of adherent platelets of each group were counted. From this result, we could draw the conclusion that collagen sponges induced the adherent of platelets dramatically, and the strongest effect could be seen in uncross-linked collagen sponges (Fig 8D).

These results suggested that the sponges fabricated by jellyfish collagen were hemocompatible. RBCs’ adhesion and aggregation in the process of blood coagulation suggested that the hemostatic mechanism of the collagen sponges was mainly due to the excellent blood absorbing property, and therefore, the concentration of hemocytes and platelets was increased to achieve a fast hemostasis. It was reported that collagen was a major activator of platelets response after injury [81]. However, the jellyfish collagen sponge, as mentioned above, could not induce activation of platelets (Figs 7C and 8D). This reflected that the contribution of jellyfish collagen to coagulation is an indirect effect due to its water absorbing property.

### 3.5 Hemostatic properties of collagen sponges

#### 3.5.1 Whole blood clotting experiment

**Fig 9:**
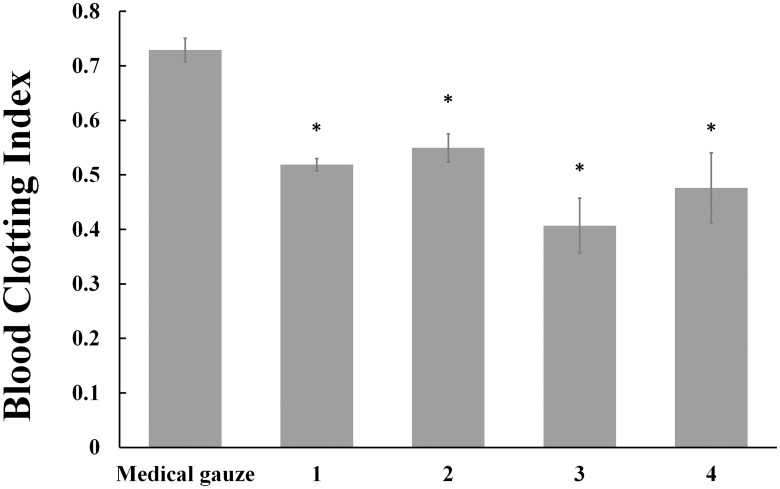
Results of whole blood clotting experiment. The clotting formation capacity is expressed as blood clotting index (BCI), which reflect the free RBCs not trapped in clots. Lower BCI value means better clotting formation capacity. Control: medical gauze; (1) uncross-linked 2.5 mg/ml collagen sponges; (2) 2.5 mg/ml collagen sponges cross-linked with 100 mM EDC for 24h; (3) uncross-linked 3.3 mg/ml collagen sponges; (4) 3.3 mg/ml collagen sponges cross-linked with 100 mM EDC for 24h. The symbol (*) denotes statistical significance from control group (*P* < 0.05).

The *in vitro* hemostatic properties of collagen sponges were evaluated by whole blood clotting experiment. Calcified rabbit whole blood was allowed to contact with collagen sponges for 5 min. Then the RBCs not trapped in clots were hemolyzed with 25 ml water. The absorbance at 540 nm can reflect the concentration of hemoglobin and the amount of free RBCs. Lower BCI value indicates a better blood clotting capability. BCI values of medical gauze group, uncross-linked 2.5 mg/ml collagen sponges, uncross-linked 3.3 mg/ml collagen sponges were 0.73 ± 0.02, 0.52 ± 0.01 and 0.41 ± 0.05 respectively (S3 Table). From Fig 9, it is obvious that compared with medical gauze group, the BCIs of collagen sponges were significantly decreased (*P* < 0.05) and the concentration of collagen also had an influence on the hemostatic property (*P* < 0.05). The hemostatic mechanism of collagen sponge is that collagen fibres can provide a physical matrix to promote platelets adhesion [38], clotting factors binding [82], and lead to clot formation rapidly [83]. Moreover, the blood clotting capability of collagen sponges will be increased as more fibres exist.

Fig 9 also showed that BCI values of sponges increased after cross-linking, and it indicated that cross-linking process could improve the hemostatic property of collagen sponges. This can be explained that the cross-linked collagen sponges which have lower levels of water absorption capability can decrease local concentration of platelets and RBCs compared with uncross-linked collagen sponges.

#### 3.5.2 Rat tail amputation experiment

**Fig 10:**
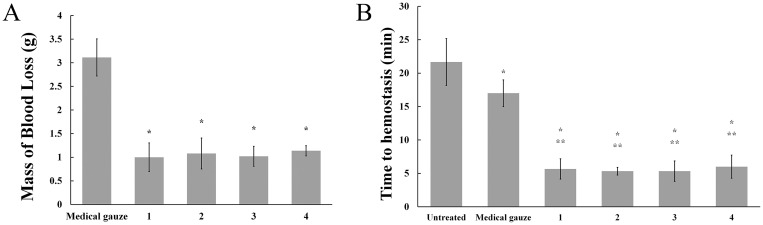
Hemostatic effect of collagen sponges in a murine tail amputation model. (A) Blood loss after tail amputation was conducted in 5 min. The symbol (*) denotes statistical significance from control group (*P* < 0.05). (B) Coagulation time of tail amputation experiment. The symbol (*) denotes statistical significance from blank (*P* < 0.05). The symbol (**) denotes statistical significance from control (*P* < 0.05). In the picture, (1) uncross-linked 2.5 mg/ml collagen sponges; (2) 2.5 mg/ml collagen sponges cross-linked with 100 mM EDC for 24h; (3) uncross-linked 3.3 mg/ml collagen sponges; (4) 3.3 mg/ml collagen sponges cross-linked with 100 mM EDC for 24h.

In order to investigate hemostatic property of collagen sponges *in vivo*, tail amputation experiment was conducted on SD rats. After applying the medical gauze, external bleeding continued for at least 15 min. Meanwhile, bleeding stopped after treated with collagen sponges within 5 min. In comparison with medical gauze, the mass of blood loss in tail amputation rat models treated with collagen sponges decreased from 3.11 g to about 1 g (Fig 10A). Fig 10B exhibited the time to hemostasis in tail amputation models of collagen sponges vs. a gauze control. The average time to achieve hemostasis of tail amputation models treated with collagen sponges was 16 min faster than that of untreated models and 12 min faster than that of models treated with medical gauze. The results showed that collagen sponges had an improved hemostatic ability with decreasing in time to hemostasis and the mass of blood loss in tail amputation rat models compared to a gauze control. A possible reason is that when the collagen sponges were applied on wound surface, the blood was absorbed rapidly by porous collagen sponges due to their higher WAC and the platelets or blood cells aggregation on the surface of sponges. Therefore, the local concentration of platelets increased in a short time and promoted the hemostatic process.

In contrast to that, there is no significant difference in time to hemostasis and the mass of blood loss in tail amputation rat models after treatment with different collagen sponges. It can be explained that the wound surface area of tail amputation rat models was small and collagen sponges can control bleeding rapidly and effectively. Differences in physicochemical properties (such as porosity and water absorption capacity) of collagen sponges did not influence on their hemostatic ability in this study.

## 4. Conclusion

In this study, we have shown that the collagen isolated from jellyfish species *R*. *esculentum* was similar to collagen type I. Porous collagen hemostatic sponges with an interconnected network pore configuration were prepared by lyophilization and subsequent chemical cross-linking. Whole blood clotting experiment indicated that collagen sponges accelerated the hemostatic process. Hemocyte morphology and adhesion test revealed that the hemostatic mechanism of the collagen sponges was mainly physical absorption. Moreover, when applied to the rat tail amputation experiment models, all collagen sponge groups showed decrease in time to hemostasis and the mass of blood loss as compared to medical gauze, which can be attributed to porous structure and the higher water absorption rate of collagen sponge. Considering the physicochemical properties and hemostatic ability of jellyfish collagen sponge, it is a suitable candidate for wound dressing applications.

## Supporting Information

S1 Table Experiment and data set of WAC of the jellyfish collagen sponges. (DOCX)

S2 Table Experiment and data set of WAC of the jellyfish collagen sponges. (DOCX)

S3 Table Experiment and data set of Whole blood clotting. Control: medical gauze; (1) uncross-linked 2.5 mg/ml collagen sponges; (2) 2.5 mg/ml collagen sponges cross-linked with 100 mM EDC for 24h; (3) uncross-linked 3.3 mg/ml collagen sponges; (4) 3.3 mg/ml collagen sponges cross-linked with 100 mM EDC for 24h.
(DOCX)
