# Supplementary material for: Isolation, Characterization and Evaluation of Collagen from Jellyfish Rhopilema esculentum Kishinouye for Use in Hemostatic Applications
Source: PLoS One. 2017 Jan 19;12(1):e0169731. doi: 10.1371/journal.pone.0169731 (PMC5245839; doi:10.1371/journal.pone.0169731)
Supplement: S1 Table — (DOCX) [file pone.0169731.s001.docx]

**Supporting Information**

**S1 Table. Experiment and data set of WAC of the jellyfish collagen sponges**

|  | Group | W_d_ | W_s_ | WAC | Average | S |
| --- | --- | --- | --- | --- | --- | --- |
| Medical gauze | 1 | 0.354 | 1.025 | 1.89548 | 2.680491 | 0.858594 |
|  | 2 | 0.308 | 1.416 | 3.597403 |  |  |
|  | 3 | 0.319 | 1.132 | 2.548589 |  |  |
| 2.5 mg/ml | 1 | 0.204 | 5.341 | 25.18137 | 27.10387 | 1.90041 |
|  | 2 | 0.215 | 6.446 | 28.9814 |  |  |
|  | 3 | 0.215 | 6.052 | 27.14884 |  |  |
| 3.3 mg/ml | 1 | 0.233 | 11.496 | 48.33906 | 40.35251 | 7.944979 |
|  | 2 | 0.209 | 6.991 | 32.44976 |  |  |
|  | 3 | 0.227 | 9.368 | 40.26872 |  |  |
