# Supplementary material for: Isolation, Characterization and Evaluation of Collagen from Jellyfish Rhopilema esculentum Kishinouye for Use in Hemostatic Applications
Source: PLoS One. 2017 Jan 19;12(1):e0169731. doi: 10.1371/journal.pone.0169731 (PMC5245839; doi:10.1371/journal.pone.0169731)
Supplement: S2 Table — (DOCX) [file pone.0169731.s002.docx]

**Supporting Information**

**S2 Table. Experiment and data set of WAC of the jellyfish collagen sponges**

|  | Group | W_d_ | W_s_ | WAC | Average | Stdev |
| --- | --- | --- | --- | --- | --- | --- |
| Uncross-linked | 1 | 0.151 | 7.454 | 48.36423841 | 40.27208048 | 8.197295295 |
|  | 2 | 0.108 | 3.244 | 29.03703704 |  |  |
|  | 3 | 0.147 | 6.529 | 43.41496599 |  |  |
| 50/12 | 1 | 0.112 | 4.241 | 36.86607143 | 35.09478927 | 4.544406118 |
|  | 2 | 0.177 | 7.179 | 39.55932203 |  |  |
|  | 3 | 0.156 | 4.658 | 28.85897436 |  |  |
| 100/12 | 1 | 0.142 | 4.181 | 28.44366197 | 29.53348785 | 0.7905496 |
|  | 2 | 0.109 | 3.364 | 29.86238532 |  |  |
|  | 3 | 0.197 | 6.165 | 30.29441624 |  |  |
| 50/24 | 1 | 0.155 | 4.295 | 26.70967742 | 22.38894009 | 3.556037954 |
|  | 2 | 0.175 | 4.105 | 22.45714286 |  |  |
|  | 3 | 0.183 | 3.477 | 18 |  |  |
| 100/24 | 1 | 0.137 | 2.681 | 18.56934307 | 19.78724341 | 0.866513461 |
|  | 2 | 0.146 | 3.141 | 20.51369863 |  |  |
|  | 3 | 0.122 | 2.596 | 20.27868852 |  |  |
