# Supplementary material for: Isolation, Characterization and Evaluation of Collagen from Jellyfish Rhopilema esculentum Kishinouye for Use in Hemostatic Applications
Source: PLoS One. 2017 Jan 19;12(1):e0169731. doi: 10.1371/journal.pone.0169731 (PMC5245839; doi:10.1371/journal.pone.0169731)
Supplement: S3 Table — Control: medical gauze; (1) uncross-linked 2.5 mg/ml collagen sponges; (2) 2.5 mg/ml collagen sponges cross-linked with 100 mM EDC for 24h; (3) uncross-linked 3.3 mg/ml collagen sponges; (4) 3.3 mg/ml collagen sponges cross-linked with 100 mM EDC for 24h. (DOCX) [file pone.0169731.s003.docx]

**Supporting Information**

**S3 Table. Experiment and data set of Whole blood clotting.** Control: medical gauze; (1) uncross-linked 2.5 mg/ml collagen sponges; (2) 2.5 mg/ml collagen sponges cross-linked with 100 mM EDC for 24h; (3) uncross-linked 3.3 mg/ml collagen sponges; (4) 3.3 mg/ml collagen sponges cross-linked with 100 mM EDC for 24h.

| VALUE | D_s_ | | | | | D_0_ |
| --- | --- | --- | --- | --- | --- | --- |
|  | Control | 1 | 2 | 3 | 4 |  |
| 1 | 1.338 | 0.941 | 1.027 | 0.692 | 0.932 | 1.842 |
| 2 | 1.34 | 0.956 | 0.996 | 0.735 | 0.862 | 1.78 |
| 3 | 1.302 | 0.934 | 0.976 | 0.793 | 0.804 | 1.835 |
| Average | 1.326667 | 0.943667 | 0.999667 | 0.74 | 0.866 | 1.819 |
| D_s_/D_0_ | 0.729338 | 0.518783 | 0.549569 | 0.406817 | 0.476086 |  |
| Stdev | 0.021385 | 0.01124 | 0.025697 | 0.050685 | 0.064094 |  |
